# Developing an Effective and Durable Film for Marine Fouling Prevention from PDMS/SiO_2_ and PDMS/PU with SiO_2_ Composites

**DOI:** 10.3390/polym14204252

**Published:** 2022-10-11

**Authors:** Jirasuta Chungprempree, Jitima Preechawong, Manit Nithitanakul

**Affiliations:** 1 The Petroleum and Petrochemical College, Chulalongkorn University, Bangkok 10330, Thailand; 2 Center of Excellence on Petrochemical and Materials Technology, Bangkok 10330, Thailand

**Keywords:** antifouling, polydimethylsiloxane elastomer, polyurethane, SiO_2_ composite

## Abstract

Polymer film coating with a highly hydrophobic surface property is a practical approach to prevent fouling of any structures in the marine environment without affecting marine microorganisms. The preparation of a polymer coating, from a simple and easy method of solution blending of hydrophobic polydimethylsiloxane elastomer and hydrophilic polyurethane with SiO_2_, was carried out in this study, with the aim of improving characteristics, and the coating demonstrated economic feasibility for antifouling application. Incorporation of SiO_2_ particles into PDMS and PDMS/PU polymer film improved mechanical properties of the film and the support fabrication of micropatterns by means of a soft lithography process. Observations from field emission scanning electron microscope (FESEM) of the PDMS/SiO_2_ composite film revealed a homogeneous morphology and even dispersion of the SiO_2_ disperse phase between 1–5 wt.%. Moreover, the PDMS film with 3 wt.% loading of SiO_2_ considerably increased WCA to 115.7° ± 2.5° and improved mechanical properties by increasing Young’s modulus by 128%, compared with neat PDMS film. Additionally, bonding strength between barnacles and the PDMS film with 3 wt.% of SiO_2_ loading was 0.16 MPa, which was much lower than the bonding strength between barnacles and the reference carbon steel of 1.16 MPa. When compared to the previous study using PDMS/PU blend (95:5), the count of barnacles of PDMS with 3 wt.% SiO_2_ loading was lower by 77% in the two-week field tests and up to 97% in the eight-week field tests. Subsequently, when PDMS with 3 wt.% SiO_2_ was further blended with PU, and the surface modified by the soft lithography process, it was found that PDMS/PU (95:5) with 3 wt.% SiO_2_ composite film with micropatterns increased WCA to 122.1° ± 2.9° and OCA 90.8 ± 3.6°, suggesting that the PDMS/PU (95:5) with 3 wt.% SiO_2_ composite film with surface modified by the soft lithography process could be employed for antifouling application.

## 1. Introduction

Generally, biofouling is formed due to the attachment of microorganisms (bacteria, algae, and fungi) and macroorganisms (barnacles, sponges, seaweed, etc.) to the surface that is covered with water [1], which is one of the major causes of physical damage to surfaces of marine structures and equipment. Biofouling has been, and continues to be, a global issue owing to its substantial environmental and economic effects. For example, the major issues caused by marine fouling in terms of economic effects are the increase in fuel consumption, of up to 40%, and overall expenses, of up to 77%, from the increase in hydrodynamic drag. These increased costs arise from increasing weight, and reducing ship speed, due to the attached organisms. Costs also arise out of corrosion. Furthermore, there are the environmental effects, such as the introduction of non-native species into local ecosystems [2]. The estimated cost of transportation delays, hull repair, cleaning and general maintenance are estimated to be about $150 billion per year [3]. As a result, industries are paying very close attention to the finding of solutions for this issue.

The fouling process typically involves the formation of biofouling on the surface of materials, which is caused by the adsorption of organic and inorganic macromolecules immediately after immersion, resulting in the formation of a conditioning film. Then, microbial cells are rapidly transported to other surfaces and form a microbial film on the surface. After that, the development of a more complex community of multicellular species means the biofilm develops with a chemical oxygen gradient and corrosion processes occur on the surface. Antifouling (AF) coatings have grown in popularity as appropriate substitutes for the standard metals with decreased corrosion effects. The antifouling mechanism adopts several methods to reduce the amount of fouling on the surfaces, such as physical and chemical methods. The chemical method focuses on biocide release and the physical method focuses on surface modification to reduce the attachment of foulants to the surface. Paints containing dispersed biocides (e.g., arsenic and mercury oxide) were the major focus for marine AF applications in the mid-nineteenth century [1]. Later, the prohibition of organo-mercury and organo-arsenic chemicals, motivated by mounting safety and health concerns, meant a decline in the use of these paints. Consequently, tributyltin (TBT) was introduced as an antifouling agent. Tributyltin was one of the most widely used chemicals for antifouling paints, based on leaching biocide. Nevertheless, environmental concerns about TBT were raised in the late 1980s, when oysters showed considerable shell thickening, and some marine creatures became locally extinct (e.g., Nucella), owing to their inability to reproduce as a result of the coating. Tin bioaccumulation was discovered in fish, seals and even ducks [4]. In 2003, the international maritime organization (IMO) banned tributyltin from antifouling application [5]. Since then, antifouling paints have been developed using copper oxide pigment, thiocyanate, cuprous bromide and so on. Copper oxide was chosen over other pigments because it was less costly and easily dispersed. However, it has been proven to have bioaccumulation and biomagnification effects on blue mussels [6].

Non-toxic, non-biocide-release based approaches for antifouling coatings aims to modify hydrophobic surfaces with nano and micro patterns to create rough surfaces. Surface roughness plays an important role in hydrodynamic performance of antifouling (AF) coatings and influences the settlement behavior of fouling, because the surface architecture of rough surfaces reduces available contact area and the ability for water droplets to adhere to the surface, due to the stable air cushion entrapped at liquid/solid interfaces, leads to higher hydrophobicity, or lower surface energy, which significantly minimizes biofouling [7]. So, the attached and adhering foulants are more easily removed from the surfaces at low hydrodynamic shears, such as the external force on ship movements, currents and waves. On the other hand, barnacles on the surface of untreated metal form biofilm and rapidly grow on the metal surface. Finally, biofilm develops with a chemical oxygen gradient, with the innermost layer in contact with the metal exhibiting an anoxic environment and the most superficial layer retaining large concentrations of oxygen. At this stage, anaerobic microorganisms, associated with metal corrosion processes, are dominant [8]. Regarding hydrophobic polymers, polydimethylsiloxane (PDMS) is one of the non-toxic and inexpensive polymers widely studied for antifouling coating [9,10]. It is preferred because the main chains of PDMS consist of siloxane bonds and side chains of methyl groups. The high bond energy and bond angle of the siloxane bond structures offer superior heat stability and flexibility for PDMS. The side chains of the methyl groups are hydrophobic (water contact angle (WCA) 107°–110°) and non-polar, leading to low surface energy and excellent fouling release [11]. Furthermore, because of its low shrinkage rates and ease of penetration into micropatterns, PDMS is suited for the soft lithography process [12]. The soft lithography process is a simple, cost-effective and scalable method for modifying PDMS surfaces into highly hydrophobic surfaces or superhydrophobic surfaces (WCA > 150°) with micro- and nano-patterns, or nanostructures, such as the surface of a lotus leaf, for antifouling application and corrosion resistance on metal surfaces [13,14,15]. However, PDMS does not have high enough mechanical properties, and when the PDMS surface was modified with micropatterns using the soft lithography process, often the micropatterns would collapse [9]. Inorganic materials, such as alumina (Al_2_O_3_), titanium dioxide (TiO_2_), zirconium (ZrO_2_), silver (Ag) and silica (SiO_2_) could be used to improve the mechanical properties, roughness and antifouling of the polymer surface [16]. Although each nanoparticle is highly efficient, pricing limits are a challenge in the industry [17]. Silica (SiO_2_) is one of the most convenient and widely utilized materials, due to its being inexpensive, having inert reactivity, well-known chemical characteristics, good mechanical properties and a mature preparation technique. Moreover, the modulus values of the PDMS elastomers are enhanced as an increasing amount of silica is introduced [18].

Although hydrophobic surfaces, such as the PDMS coating, have shown high water repellence and potential against many marine organisms, oils found in marine environments attach to the surface of the coating, thereby reducing efficiency and performance of the polymer coating, and allowing slime composed of diatoms and bacterial attachments, which limits the usage of polymer coating in practical applications. Consequently, although the hydrophobic characteristic is the most important factor for polymer coating for antifouling applications, amphiphobic surface polymers, which have both hydrophilic and hydrophobic properties, have piqued interest due to their unique wetting behavior and potential applications [19]. Thus, to improve the amphiphobic surface properties of the coating, various techniques have been employed, such as inducing a hierarchical surface, and grafting or blending with a hydrophilic polymer. In 2018, Bhalani and co-workers prepared poly(vinyl pyrrolidone) and induced hierarchical surface morphology on a poly(vinylidene fluoride) membrane with the prepared poly(vinyl pyrrolidone) to obtain a material with superhydrophilic and underwater oleophobic characteristics [20]. Bhalani et al. reported the preparation of low toxic polymers for antifouling using a blended membrane of PVDF-blend from poly(vinylidene fluoride) and poly(methyl methacrylate)-co-poly(chloromethyl styrene). The modified membranes, obtained by covalent functionalization of PVDF, that were prepared had increased hydrophilicity and decreased protein fouling properties [21]. However, grafting and hierarchical surface modification required a variety of chemicals and it was a very complicated method. Polymer blending is one of the best solutions for producing antifouling materials, since it is a less expensive and quicker means to induce surface enrichment with unique properties [22]. It involves the combination of two or more polymers to give applications that benefit from the excellent properties of each polymer. A blend of different quality polymers has made it more cost-effective to create novel materials with the desired qualities, mechanical properties, abrasive resistance, etc. [23]. Polyurethane has a wide range of applications, such as its use in biomedical applications, the automotive industry, building, textiles and others, due to its non-toxic, non-flammable, environment-friendly characteristics, and its good mechanical properties, economical manufacturing process and wide range of other properties [24,25]. In addition, many articles have reported on polyurethane modification for decreased protein adsorption and cell adhesion [26].

In our previous study, a highly hydrophobic surface polymer coating for antifouling application made from PDMS/PU blends was prepared and characterized. In this study, polymer coatings for antifouling application from PDMS with different amounts of SiO_2_ were prepared and studied. Mechanical properties and hydrophobicity of PDMS composite films were improved with the addition of different amounts of SiO_2_. This was aimed at presenting a simple procedure for preparing nontoxic material and an easily processable polymer coating for antifouling. Subsequently, PDMS with 3 wt.% SiO_2_ was further blended with PU. The performances of the PDMS/SiO_2_ and PDMS/PU (95:5) with SiO_2_ composite and PDMS/PU (95:5) with SiO_2_ composite modified surface with soft lithography process were studied in terms of increased mechanical and surface properties.

## 2. Materials and Methods

### 2.1. Materials and Chemicals

The polydimethylsiloxane elastomer (PDMS) employed in this study had a density of 1.03 and was prepared from silicone elastomer kit Dow Corning Midland Michigan USA, supplied by Dow Corning, under the tradename Sylgard 184. The silicone base and a curing agent were contained in a two-part chemical (Part A and Part B). Part A included dimethyl siloxane, whereas Part B contained dimethyl, methylhydrogen siloxane, dimethyl siloxane, dimethylvinyl terminated, dimethylvinylated and trimethylated silica, tetramethyl tetravinyl cyclotetra siloxane and ethyl benzene. Smooth-On, USA, supplied the polyurethane (PU) which was used in this study, and which had a density of 1.036 and was marketed as Smooth-On 204. The SiO_2_ particle used in this investigation was provided by Chemipan, Bangkok, Thailand under the tradename of CAB-O-SIL. The SiO_2_ particle size was approximately 0.2–0.3 µm and had a refractive index of 1.46. The SiO_2_ particle had a hydrophilic surface with a surface area of 199 m^2^/g.

### 2.2. Preparation

#### 2.2.1. Preparation of PDMS/SiO_2_ Composite

In this study, the preparation of PDMS with silica composites employed a procedure outlined by Liu, Junshan et al., 2015. Silica particles were prepared by mixing with toluene and the mixture was magnetically stirred and ultrasonically oscillated to enhance dispersion of the particles [27]. Next, PDMS was separately dissolved in toluene and mechanically stirred at 250 rpm for 1 h. After that, the PDMS solution was added to the SiO_2_ suspension with varying amounts of SiO_2_ at 0, 1, 3, 5, 7, 10 wt.% and stirred for a further 1 h and then left to evaporate in a chemical hood. Next, a curing agent was introduced into the system. Finally, the polymer composite was poured into a mold. During the process, if air bubbles would occur, a degassing step would be required. This was carried out by placing the samples in a vacuum desiccator for 30 min to remove any small and large bubbles on the surface of the sample. Next, the sample was cured by heating in an oven at 60 °C for 3 h. Finally, after cooling, the obtained samples were removed from the mold.

#### 2.2.2. Preparation of the PDMS/PU (95:5) with SiO_2_ Composite

Polyurethane (PU) was prepared with a mixing ratio of 100:90 by weight of part A and part B. The PDMS/PU with SiO_2_ composite, as shown in Table 1, was prepared by mixing with toluene and the mixture was magnetically stirred and ultrasonically oscillated to enhance dispersion of the particles. After removing the solvent from the PDMS/SiO_2_ composite, PU was added and further mixed for 10 min using a mechanical stirrer at 250 rpm. Next, the curing agent was introduced at a weight ratio of 10:1. Finally, the polymer composite was poured into a mold or a soft lithography mold. Any air bubbles that might occur throughout the procedure, necessitated a degassing step in which the samples would be placed in a vacuum desiccator for 30 min to eliminate any tiny and big bubbles on the sample’s surface. After that, the sample was cured for 3 h at 60 °C in an oven. Finally, the obtained samples were removed from their molds after cooling [10].

### 2.3. Characterization of the PDMS/SiO_2_, PDMS/PU with SiO_2_ Composites

#### 2.3.1. Scanning Electron Microscope (SEM)

A field emission scanning electron microscope (JEOL, Hitachi, Model S-4800, Tokyo, Japan) was used to study the phase morphology of cross-sections of the composite films. The composite film samples were prepared by submerging them in liquid nitrogen for 3–5 min. The samples were then broken by gripping on both sides. Following that, the specimens were coated with platinum under vacuum before being observed by using a 2 kV accelerating voltage. Material identification and phase dispersion of the samples were also investigated using SEM/EDX.

#### 2.3.2. Contact Angle Measurement

Surface characteristics of the prepared composites were investigated using water contact angle and oil contact angle measurements. The water contact angles and oil contact angles were observed by a Krȕss, Hamburg, Germany (model DSA 10) contact angle measuring instrument at ambient temperature. Using a micro-syringe, a 10 µL sessile droplet of deionized water or castor oil (supplied by Hong Huat Co., Bangkok, Thailand) was vertically placed onto the surface of the composite film. A camera with a magnifying lens was used to capture photographs of the water drop or oil drop formation on the surface of the film.

#### 2.3.3. Mechanical Test (LLOYD)

Mechanical characteristics of the PDMS/SiO_2_ composites and PDMS/PU with SiO_2_ composite films were assessed using a universal testing machine with the procedure outlined in ASTM D882 standards. The stretching speed was 50 mm/min, and the dimensions of the samples were 100 mm × 10 mm × 1 mm. An average result was collected from five individual specimens for each sample.

#### 2.3.4. Microfouling Analysis

Microfouling analysis of the samples was carried out by field tests in the Gulf of Thailand, at Koh Sichang Marine Science Research Centre of Chulalongkorn University (13°09′10.6″ N 100°49′02.6″ E). All samples were installed on Teflon frames for up to eight weeks over the period of April to June to determine the level of microfouling. This time period was chosen because it is a period in which there is a high barnacle population because of breeding and broadcast-spawning in the warmer seawater temperatures [28]. The reference specimen used in this study was SS400 carbon steel, which is typically used for general structural purposes, and it also commonly used for marine ship structures, high-rise buildings, and so on [29]. The size of the Teflon frame was 0.6 × 0.6 m^2^. The frames were set at a depth of 3.0 m from the seawater level [30]. The average dimensions of barnacles, i.e., between 5 to 20 mm in diameter and at least 30 mm in heigh, were selected for this experiment. The base area of the barnacle, A, (square meters) was approximated from the average base diameter, $d_{0}^{2}$, (meters). The base area of the barnacle was calculated according to Equation (1). Barnacle adhesion force measurements were performed using a digital force gauge (Inspex IPX-808) at room temperature for all samples when barnacles settled on the surface. The capacity of the force measuring device was between 0 to 150 N (0 to 34 lb) to an accuracy of ±0.5 N (±0.1125 lb). Furthermore, according to ASTM D5618-94, the adhesion strength, τ, (pascal, Pa) of the fouling release surfaces was evaluated by measuring the shear force, F, (newton, N) required to remove the barnacle by the base area, A, (square meters) of the barnacle, according to Equation (2).

(1) $$A=\frac{1}{4}\pi d_{0}^{2}$$

(2) $$\tau =\frac{F}{A}$$

#### 2.3.5. Atomic Force Microscopy (AFM)

With nanoscale spatial and piconewton force resolution, atomic force microscopy (AFM, Bruker, Karlsruhe, Germany) accurately measures characteristics of materials and inter-material interactions. The AFM photodetector’s spatial sensitivity was measured against a clean silicon wafer. The topology and elastic modulus of each sample were measured from five separate locations with sizes of 1 × 1 × 0.1 cm using the scanAsyst-Air probe (silicon tip radius < 10 nm) in PeakForce QNM modes from Bruker with a spring constant of 0.4 N/m. Peak Force Quantitative Nanomechanical Mapping (PFQNM), also known as quantitative nanomechanical mapping, is a semi-contact AFM mode that generates both height and phase images. The phase image recognized modulus differences.

## 3. Results and Discussion

### 3.1. PDMS with SiO_2_ Composite

#### 3.1.1. Morphology of the PDMS/SiO_2_ Composite

**Figure 1:**
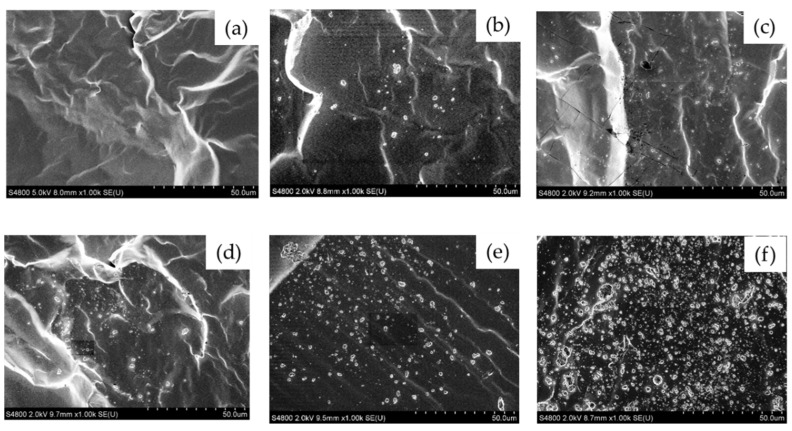
SEM image (×1.00k) of cross-section view of (**a**) neat PDMS; (**b**) PDMS with 1 wt.% SiO_2_; (**c**) PDMS with 3 wt.% SiO_2_; (**d**) PDMS with 5 wt.% SiO_2_; (**e**) PDMS with 7 wt.% SiO_2_; and (**f**) PDMS with 10 wt.% SiO_2_.

The morphologies of neat polydimethylsiloxane (PDMS), and polydimethylsiloxane (PDMS) composite, with PDMS as the matrix phase and SiO_2_ as the disperse phase, were observed using field-emission scanning electron microscopy (FE-SEM), and are shown in Figure 1. The rough surface of the cross-section, as shown in neat PDMS, indicated the ductile characteristic of the material [31]. Compared with the neat PDMS surface, the PDMS composite surface, with SiO_2_ particles from 1 to 10 wt.%, showed microroughness, which correlated positively with filler content [32]. The dispersion of particles inside the matrix was more uniform and homogeneous from 1 to 5 wt.% SiO_2_. The average diameter of dispersed SiO_2_ particles was about 0.2–0.3 µm. The quantity of SiO_2_ particle agglomerations and their size increased as the silica loading increased. Particle agglomeration occurred when the amount of SiO_2_ increased to 7 wt.% and 10 wt.% by weight, as shown in the SEM image (see Figure 1). It was also demonstrated that with increased wt.% of SiO_2_ particle loadings, the probability of bulk formation increased, resulting in a loss of material properties owing to the heterogeneous dispersion of silica particles in the matrix [33,34].

#### 3.1.2. Water Contact Angle (WCA) of the PDMS/SiO_2_ Composite

**Figure 2:**
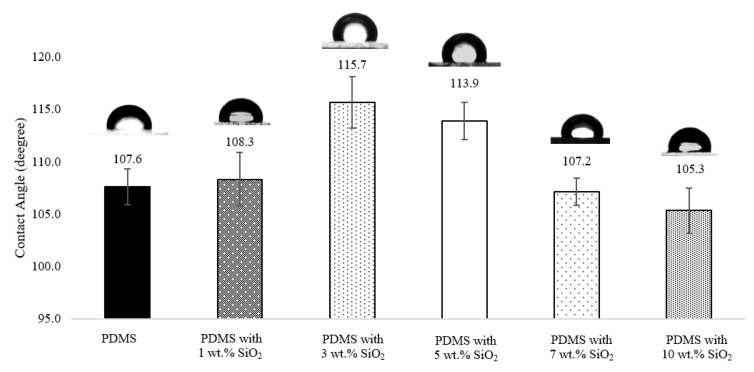
Water contact angle on various material surfaces of neat PDMS; PDMS with 3 wt.% SiO_2_; PDMS with 5 wt.% SiO_2_; PDMS with 7 wt.% SiO_2_ and PDMS with 10 wt.% SiO_2_.

Water contact angle (WCA) was used to evaluate the hydrophobic surface characteristic, based on surface wettability of the PDMS and PDMS/SiO_2_ composites. To achieve antifouling properties, the surface wettability of the material, determined by water contact angle, should be greater than 90°, which is the characteristic of a hydrophobic surface. The contact angle of water droplets on neat polydimethylsiloxane surface had a value of 107.6° ± 1.7°, as shown in Figure 2. The addition of 1 wt.% of SiO_2_ gave a WCA value of the PDMS/SiO_2_ composite of 108.3° ± 2.5°, which was relatively similar to that of the neat PDMS. The addition of 1 wt.% SiO_2_ was found to have little effect on the WCA of the composite. This might have been due to the small amount of SiO_2_ added which was insufficient to reach the dispersion limit to produce a surface architecture of hierarchically rough surfaces. Similar behavior was also observed by Tapasa, K. and coworker [35]. However, WCA increased to 115.7° ± 2.5° when the ratio of SiO_2_ was 3 wt.% and slightly decreased to 113.9° ± 1.8° when the ratio of SiO_2_ was 5 wt.%. This might have been because of the synergistic impact of the surface roughness and the hydrophobic matrix [32]. The water contact angle value of the composites further decreased when wt.% SiO_2_ content increased to 7 wt.% at 107.2° ± 1.3° and 10 wt.% at 105.3° ± 2.1°, which was probably due to the agglomeration of the SiO_2_ particles. Consequently, WCA of the PDMS/SiO_2_ composites was highest when the wt.% of SiO_2_ was at 3 wt.%. From the result, adding SiO_2_ was one way to increase the hydrophobicity of the polymer surface. It was also possible that addition of SiO_2_ to the PDMS/PU blended (95:5) films would also further enhance the hydrophobic characteristics of polymer blend films, since the contact angle of water droplets on PDMS/PU blend (95:5) films in the earlier studies showed a range of WCA between 103.4° ± 3.8° to 91.4° ± 0.8° [12]. Furthermore, it is widely acknowledged that converting the hydrophobic surface of polymer film to a highly hydrophobic surface is a complex manufacturing procedure, which may involve complicated processing techniques, such as the use of plasma, etching, electrochemical reactions, decomposition etc. [36,37]. In this work, the soft lithography process was subsequently employed to further improve the hydrophobic characteristic of the materials to obtain highly hydrophobic surfaces for antifouling applications by means of a simple and cost-effective approach.

#### 3.1.3. Barnacle Measurements

**Figure 3:**
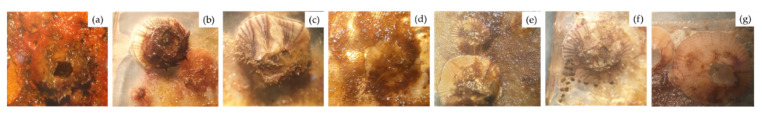
Digital microscope images of marine biofouling on various material surface in seawater (**a**) carbon steel; (**b**) neat PDMS; (**c**) PDMS with 1 wt.% SiO_2_; (**d**) PDMS with 3 wt.% SiO_2_; (**e**) PDMS with 5 wt.% SiO_2_; (**f**) PDMS with 7 wt.% SiO_2_ and (**g**) PDMS with 10 wt.% SiO_2_.

**Figure 4:**
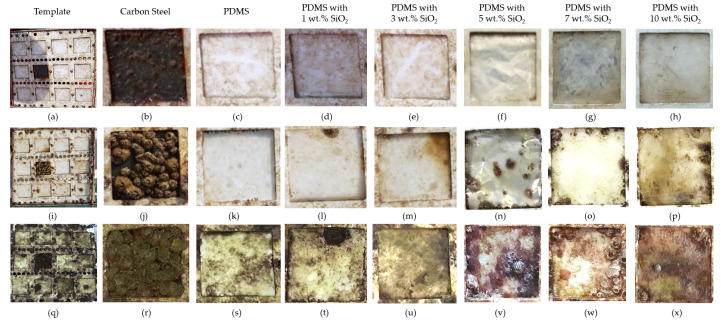
Marine biofouling on various sample surfaces after immersed in seawater environment (in April-June) (**a**–**h**) immersed for 2 weeks; (**i**–**p**) immersed for 4 weeks and (**q**–**x**) immersed for 8 weeks.

**Figure 5:**
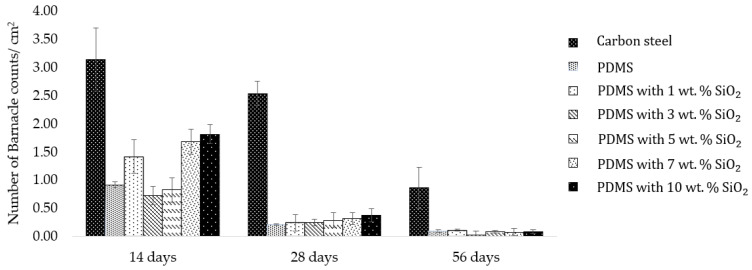
Barnacle counts after 14 days, 28 days and 56 days in seawater (facing away from the shore).

Neat PDMS and PDMS/SiO_2_ composite films were further examined in a short-term marine field test at Koh Sichang Marine Science Research Centre of Chulalongkorn University (13°09′10.6″ N 100°49′02.6″ E) in Thailand from April to June to study antifouling performance of the composite films by determining the number of barnacles and barnacle adhesion strength on the surfaces. The number of barnacles of each sample was compared with carbon steel (JIS SS400) as a reference material, using a digital microscope (Figure 3). After 2 weeks, the blank control carbon steel was fully covered by fouling organisms, including barnacles. on the surface (Figure 4). Moreover, corrosion and rusting were also observed on the surface of the carbon steel. Barnacles on the surface of carbon steel presented at approximately 3.1 ± 0.6 marine barnacles/cm^2^. After 4 and 8 weeks, this decreased from 2.5 ± 0.2 to 0.9 ± 0.4 marine barnacles/cm^2^, which was due to the increase in the size of barnacles. However, the number of barnacles of the neat PDMS in sea water after 2 weeks was approximately 0.9 ± 0.1 marine barnacles/cm^2^ for the samples facing away from the shore (Figure 5). When immersed longer, some biofoulings disappeared from the surface of the neat polydimethylsiloxane, since fouling was more easily released from the surface of the neat PDMS samples due to weak adhesion between the surfaces [38]. Furthermore, PDMS with 3 wt.% SiO_2_ exhibited slightly less barnacles than the blank control carbon steel and the neat PDMS samples. The marine organism counts decreased by 77% at 2 weeks and 97% at 8 weeks, when compared to the reference carbon steel.

**Figure 6:**
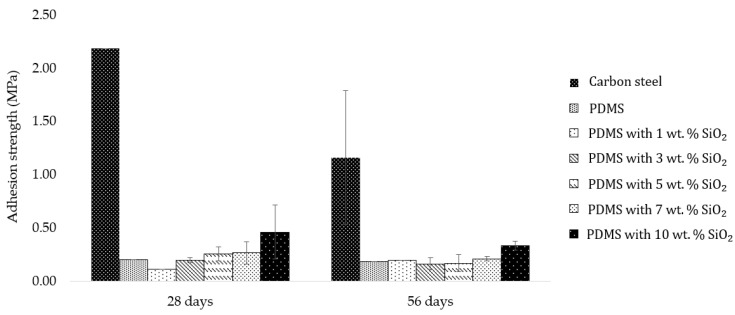
Barnacle adhesion strength (MPa) on surfaces of different materials after 28 days and 56 days in seawater (facing away from the shore).

Apart from the number of barnacles on the surface, barnacle adhesion strength, which relates to the barnacle release property, was also another significant factor linked to antifouling application that was investigated. Barnacle adhesion strength was calculated by dividing barnacle adhesion force with the barnacle base area. The result showed that the PDMS with 3 wt.% SiO_2_ composite exhibited lower barnacle adhesion strength, which was 85–90% less than barnacle adhesion strength on carbon steel (Figure 6). Furthermore, when compared to previous studies, the barnacle counts of 3 wt.% SiO_2_ and 5 wt.% SiO_2_ were lower than the barnacle counts on PDMS/PU blend (95:5) films, although barnacle adhesion strength remained rather similar [39]. From the results, surface roughness played an important role in hydrodynamic performance of the antifouling (AF) coating and influenced the settlement behavior of fouling. The surface architecture of the rough surfaces reduced available contact area and the ability for water droplets to adhere to the surface, due to the stable air cushion entrapped at liquid/solid interfaces, leading to higher hydrophobicity or lower surface energy, which significantly minimized the biofouling. So, the attached and adhering foulants were more easily removed from the surfaces at low hydrodynamic shears, such as the external forces due to the ship movement, currents and waves. It was indicated that all PDMS/SiO_2_ composite surfaces had a significantly lower number of barnacles and lower barnacle adhesion strength than carbon steel. So, it was clearly illustrated that the PDMS/SiO_2_ composite film surface improved both antifouling attachment and fouling release performance.

#### 3.1.4. Mechanical Properties Studies of PDMS/SiO_2_ Composite

**Figure 7:**
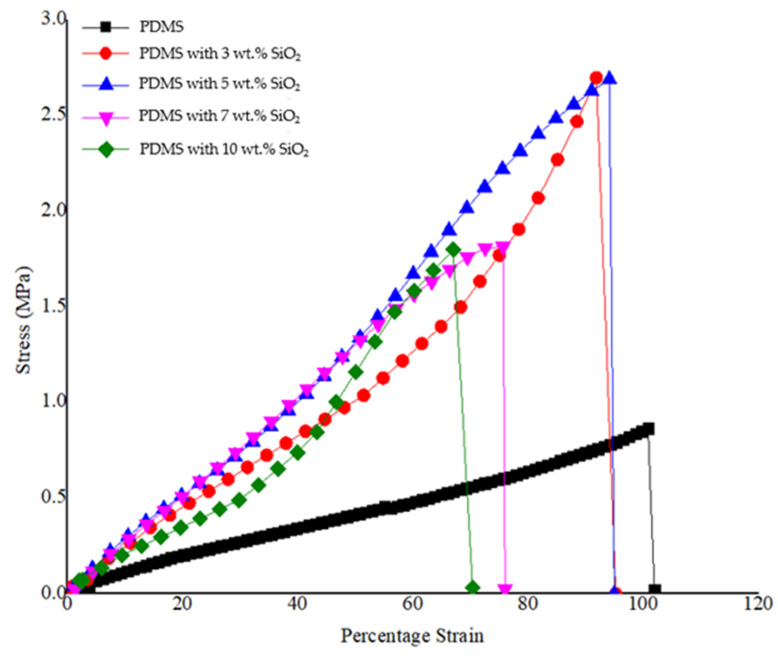
Stress–strain curves of neat PDMS; PDMS with 3, 5, 7 and 10 wt.% SiO_2_ (at room temperature).

Another important property for the application of an antifouling coating film are mechanical properties, such as tensile strength. The strength of the neat PDMS and PDMS/SiO_2_ composite films, and mechanical properties of both neat PDMS and PDMS/SiO_2_ composites were studied. The results obtained from the tensile tests for the neat PDMS and PDMS/SiO_2_ composites with various weight percentages of SiO_2_ are presented in Figure 7, which is a plot between stress and strain of the different composite films. The neat PDMS film showed an elastic property with a Young’s modulus of 1.50 ± 0.18 MPa. Young’s modulus of the composite with the addition wt.% of SiO_2_ in the PDMS matrix phase at 3, 5, 7 and 10 wt.% was 2.92 ± 0.96 MPa, 3.07 ± 0.59 MPa, 2.68 ± 1.91 MPa and 2.30 ± 0.39 MPa, respectively. It is interesting to note that further increasing of the SiO_2_ content from 7 wt.% caused SiO_2_ particles to agglomerate (as observed from SEM results), and, thus, the optimum wt.% of SiO_2_ for improving mechanical characteristics of the composites was between 3–5 wt.% [39]. These results showed that polydimethylsiloxane elastomer filled with SiO_2_, as a dispersal phase, improved the mechanical properties of the polymer film and still retained good properties of PDMS, in terms of resistance to deformation, as well as the opportunity to build microstructures using the soft lithography process [40].

#### 3.1.5. Atomic Force Microscopy (AFM) of PDMS/SiO_2_ Composite

**Figure 8:**
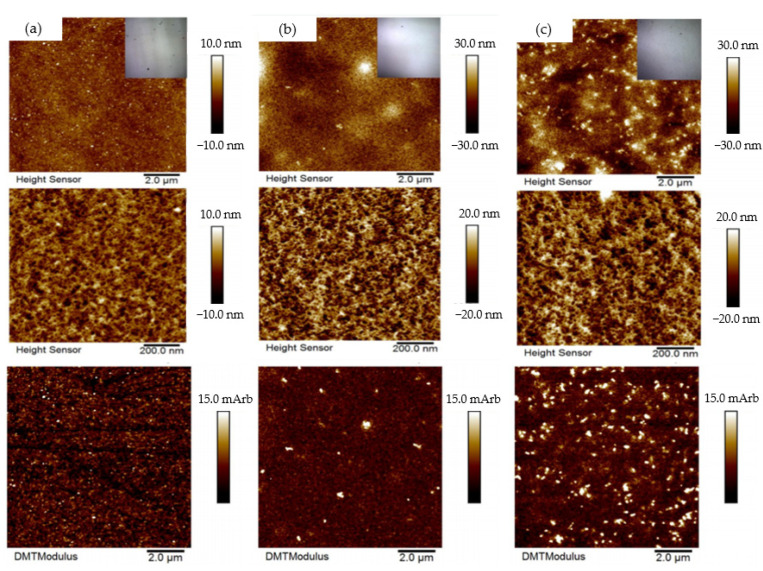
AFM topology images of (**a**) neat PDMS; (**b**) PDMS with 3 wt.% SiO_2_ and (**c**) PDMS with 5 wt.% SiO_2_.

To support WCA results, surface roughness of neat PDMS, PDMS with 3 wt.% SiO_2_ and PDMS with 5 wt.% SiO_2_ composite films were studied by means of an atomic force microscope (AFM). Surface roughness is a key factor to increase water contact angle of a surface because the surface architecture of hierarchically rough surfaces reduces available contact area and the ability for water droplets to adhere to the surface [41]. The surface roughness of the PDMS film could be further improved by introducing SiO_2_ particles and the material could become more hydrophobic after the addition of SiO_2_ particles in the PDMS matrix. AFM was employed to collect topography images and measure roughness of the scanned samples by showing a brighter phase and a darker phase, with the brighter phase showing a higher modulus than the darker phase [42]. The AFM topography images of the PDMS with SiO_2_ composite film presents the dark areas as PDMS and the bright areas as SiO_2_ (Figure 8). The neat PDMS, PDMS/SiO_2_ composite with 3 and 5 wt.% SiO_2_ exhibited surface roughness in increasing order at nanoscale polymeric structures, according to the AFM results. Figure 8 illustrates the AFM result, which indicates a roughness value of less than 10 nm for the neat PDMS and the surface roughness of the film increased by adding SiO_2_ particles. Moreover, the relative modulus of the polymeric film was also improved when 3 wt.% SiO_2_, and 5 wt.% of SiO_2_ was added to the PDMS matrix.

### 3.2. PDMS/PU with SiO_2_ Composite

#### 3.2.1. Morphologies and Compositions of the PDMS/PU with SiO_2_ Composite

**Figure 9:**
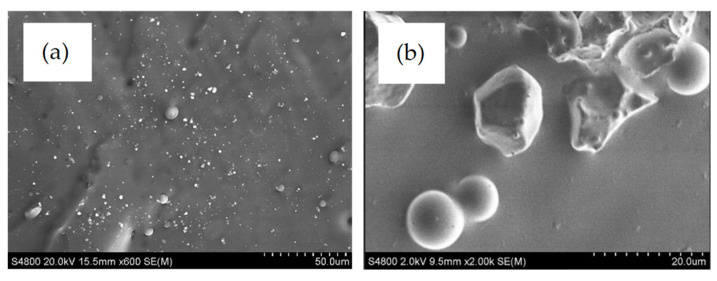
SEM image of (**a**) ×600 and (**b**) ×2k of PDMS/PU (95:5) with 3 wt.% SiO_2_ composites.

From the results of PDMS with SiO_2_ composites in the previous section, the optimum wt.% of SiO_2_ for improving the mechanical properties of PDMS was 3 wt.% of SiO_2_, so this was employed to further improve the PDMS/PU (95:5) blend film. It gave the lowest number of barnacle attachments, low bonding strength of barnacles and high contact angle of the film after fabricating with micropatterning, using the soft lithography process from previous work [10]. The main objective of using SiO_2_ particles in a low viscous liquid polymer blend was ease of the process by improving surface wettability and surface strength. The surface morphology of the PDMS/PU (95:5) with SiO_2_ composite was observed with field-emission scanning electron microscopy (FESEM). The PDMS/PU (95:5) film without SiO_2_ filler clearly illustrated homogenous distribution of PU in PDMS phase, in which the average diameter of dispersed polyurethane particles was about 8.3 ± 5.6 µm. Nonetheless, PDMS/PU (95:5) with SiO_2_ composite showed that silica particles caused decrease in size of PU droplet diameter and resulted in a finer dispersion of PU in the PDMS matrix to around 2.7 ± 1.7 µm (see Figure 9). It was confirmed that the addition of SiO_2_ filler modified and improved PU compatibility with the hydrophobic PDMS matrix [32].

**Figure 10:**
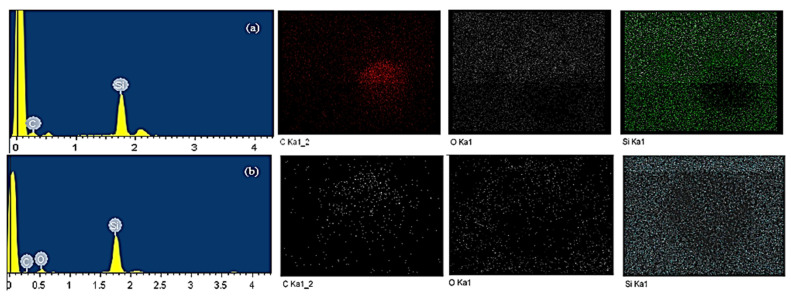
SEM-EDX image of: (**a**) PDMS/PU blend (95:5); (**b**) PDMS/PU (95:5) with 3 wt.% SiO_2_ composites.

Energy-dispersive X-ray spectroscopy (EDX) mapping spectrum of PDMS/PU (95:5) with SiO_2_ composite indicated the characteristic elemental distribution (C, O and Si) (see Figure 10). It was observed that the oxygen (O) weight percentage in the PDMS/PU with SiO_2_ composite increased from 14.24 wt.% to 31.68 wt.% and weight percentage of carbon atoms decreased from 53.47 wt.% to 32.27 wt.% with the addition of SiO_2_ into the PDMS/PU (95:5) blend. It was also illustrated that when SiO_2_ was added to the PDMS/PU blend, the silicon (Si) weight percentage of the surface increased from 32.29 wt.% to 36.05 wt.%, as shown in Table 2.

#### 3.2.2. Water Contact Angle (WCA) of the PDMS/PU (95:5) with SiO_2_ Composite

**Figure 11:**
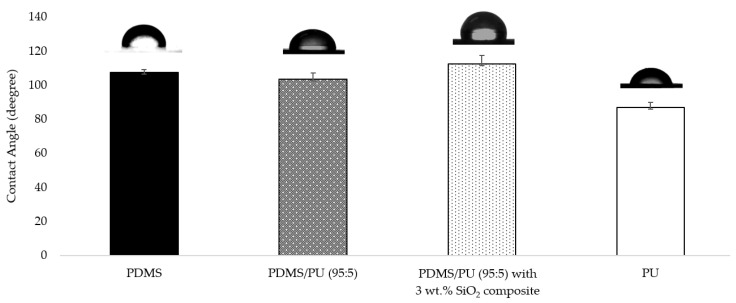
Water contact angle on various material surfaces.

The effect of the addition of SiO_2_ on the surface wettability of PDMS/PU (95:5) with SiO_2_ composite is shown in Figure 11. The surface characteristic of the neat PDMS film was hydrophobic, with a WCA value of about 107.6° ± 1.7°, and the surface of neat PU was hydrophilic, with a WCA value of about 86.9° ± 3.2°. When PDMS was blended with PU, it was discovered that the PDMS/PU blended (95:5) film showed higher WCA than the neat polyurethane film at 103.4° ± 3.8° [39]. Moreover, with the addition of the SiO_2_ in PDMS/PU (95:5) with 3 wt.% SiO_2_ composites, the water contact angle (WCA) value of the composite surface increased to 112.62° ± 4.9°. This increased WCA might have been caused by the addition of SiO_2_ particles to the polymer composite film which enhanced surface roughness of the composite, resulting in increased WCA.

#### 3.2.3. Mechanical Properties Study of the PDMS/PU (95:5) with SiO_2_ Composite

**Figure 12:**
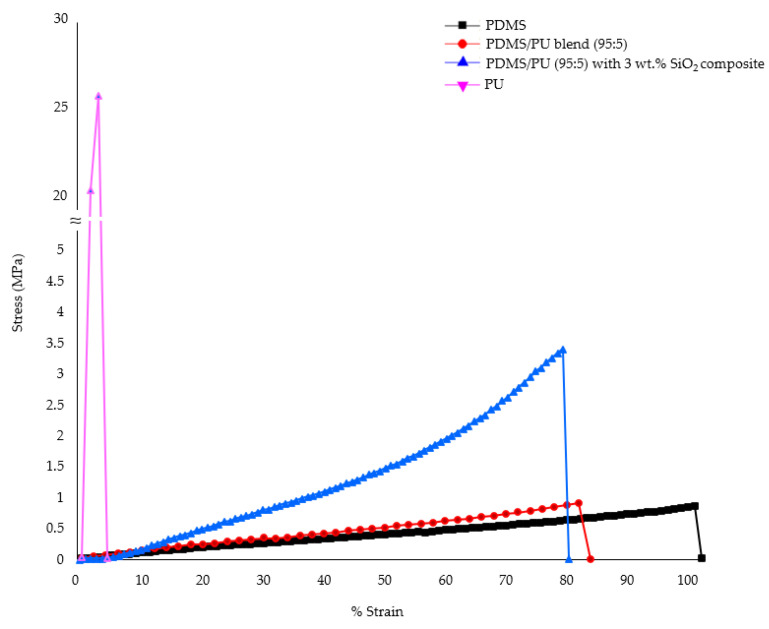
Stress–strain curves of neat PDMS film; PU film; PDMS/PU blend (95:5) film; PDMS/PU (95:5) with 3 wt.% SiO_2_ composite film.

The neat polydimethylsiloxane film demonstrated a Young’s modulus of 1.50 ± 0.18 MPa. After blending with polyurethane, mechanical properties of the PDMS/PU (95:5) polymer blend films were improved, due to the introduction of rigid polyurethane. From the graphs, it can be observed that the Young’s modulus of the PDMS/PU blend (95:5) film increased to 1.75 ± 0.35 MPa, which was superior to the neat PDMS. Subsequently, addition of 3 wt.% SiO_2_ to the PDMS/PU with SiO_2_ composite increased the Young’s modulus to twice that of the neat PDMS (see Figure 12). These results showed that PDMS/PU (95:5) with 3 wt.% SiO_2_ improved the mechanical properties of the PDMS/PU composite film making it more suitable for subsequent fabrication with the soft lithography process to impart micropatterns on the surface.

### 3.3. Characteristics of PDMS/PU (95:5) with 3 wt.% SiO_2_ Composite Film with Micropatterns Fabricated by Soft Lithography Process

#### 3.3.1. Morphology of PDMS/PU (95:5) with 3 wt.% SiO_2_ Composite Film with Micropatterns Fabricated by the Soft Lithography Process

**Figure 13:**
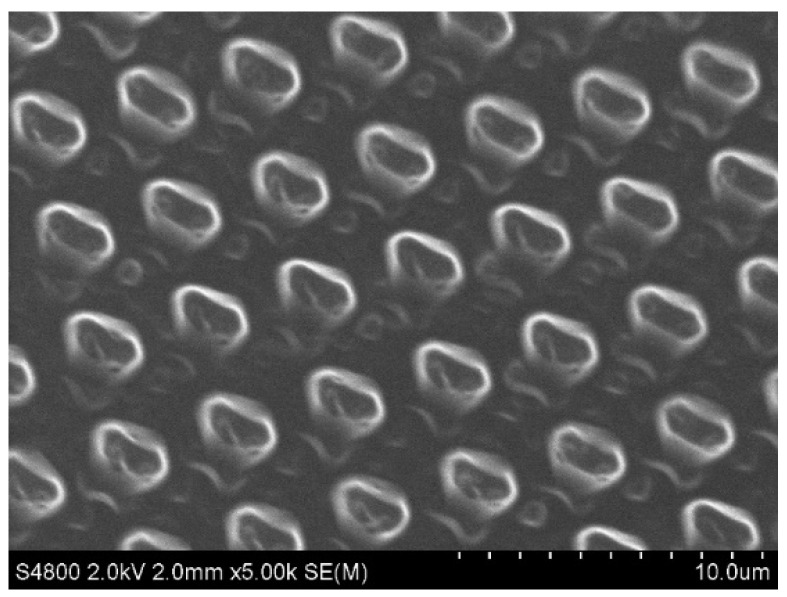
SEM image of the PDMS/PU (95:5) with 3 wt.% SiO_2_ composite film with ridge pillar patterns.

The soft lithography process was used to impart microstructures or micro patterns on the surface of PDMS/PU (95:5) with 3 wt.% SiO_2_ composite film to further improve the hydrophobic characteristics of the polymer composite film. SEM was used to examine the microstructures or micropatterns on the PDMS/PU (95:5) with 3 wt.% SiO_2_ composite surface (see Figure 13). The top view of the composite film revealed consistent pillar structures on the surface, indicating that the soft lithography process was able to further modify the PDMS/PU (95:5) with 3 wt.% SiO_2_ composite film.

#### 3.3.2. Water Contact Angle (WCA) and Oil Contact Angle (OCA) of PDMS/PU (95:5) with 3 wt.% SiO_2_ Composite Film with Micropatterns Fabricated by Soft Lithography Process

**Figure 14:**
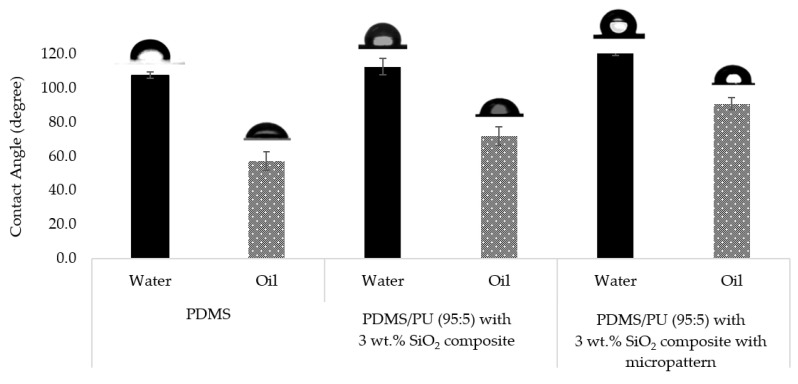
Water contact angle and oil contact angle on various material surfaces.

Surface wettability of PDMS, PDMS/PU (95:5) with 3 wt.% SiO_2_ composite film and PDMS/PU (95:5) with 3 wt.% SiO_2_ composite film with micropatterns fabricated by soft lithography process are shown in Figure 14. The surface of the neat PDMS film surface gave WCA of about 107.6° ± 1.7° and OCA of about 57.2° ± 5.4°. However, PDMS/PU (95:5) with 3 wt.% SiO_2_ composite film showed slightly higher WCA and OCA than the neat PDMS film at about 112.6° ± 4.9° and 71.7° ± 5.4°. PDMS/PU (95:5) with 3 wt.% SiO_2_ composite film with micropatterns fabricated by soft lithography process gave WCA and OCA of 122.1° ± 2.9° and 90.8° ± 3.6°. The results showed that the soft lithography process could further improve surface properties of the polymer composite film, making it more suitable for antifouling application.

## 4. Conclusions

In this work, nontoxic polymer film coatings made from PDMS with SiO_2_ and PDMS/PU with SiO_2_ composite film were successfully prepared to provide antifouling performance through a simple technique in which SiO_2_ was initially added to the PDMS matrix phase to enhance mechanical property of the film in order to support the fabrication of micro-/nano-structures by the soft lithography process. When SiO_2_ was introduced to PDMS, the phase morphology of the polymer composite clearly illustrated good distribution from 1 to 5 wt.% of SiO_2_ addition. Furthermore, at 3–5 wt.% of SiO_2_ addition, hydrophobicity and mechanical properties of the material were enhanced. More importantly, the PDMS with SiO_2_ composite film prevented fouling attachment and lower barnacle adhesion strength. The PDMS with 3 wt.% SiO_2_ composite film showed decreased adhesion strength of fouling on surface, with 85–90% lower adhesion strength than the reference carbon steel. Subsequently, SiO_2_ was introduced to the PDMS/PU (95:5) blend to produce PDMS/PU (95:5) with 3 wt.% of SiO_2_ composite film. Based on the best composite film behavior result, 3 wt.% of SiO_2_ was applied to the PDMS/PU blend (95:5) film. PDMS/PU (95:5) with 3 wt.% of SiO_2_ composite film with ridge pillar micropatterns increased water contact angle to 122.1° ± 2.9° and oil contact angle to 90.8° ± 3.6°. Moreover, the addition of 3 wt.% SiO_2_ enhanced the Young’s modulus to twice that of the neat PDMS. This study suggests a simple and easy method for the development of an environmentally safe and effective antifouling film that would be useful for the marine industry.

## Figures and Tables

**Table 1:** The formulation of PDMS/PU with SiO_2_ composite.

PDMS/PU Ratio	wt.% SiO_2_
95:5	0 wt.% SiO_2_
95:5	3 wt.% SiO_2_

**Table 2:** EDX analysis results including weight and atomic percentage of various elements found on the surface of the materials.

Element	PDMS/PU Blend (95:5)		PDMS/PU (95:5) with 3 wt.% SiO_2_	
	Weight%	Atomic%	Weight%	Atomic%
C	53.47	68.58	32.27	45.14
O	14.24	13.72	31.68	33.28
Si	32.29	17.71	36.05	21.58

## Data Availability

The authors confirm that the data supporting in this study are available within the article. Raw data that support the findings of this study are available from the corresponding author, upon reasonable request.
